# Sub-Acute Parenchymatous Hepatitis and Gastro-Enteritis1Read before the Buffalo Clinical Society.

**Published:** 1892-04

**Authors:** A. L. Benedict


### SUB-ACUTE PARENCHYMATOUS HEPATITIS AND GASTRO-ENTERITIS.[1]

By A. L. BENEDICT, A. M., M. D.

On December 17, 1891, I was called to see Mr. M., a bartender, aged twenty-eight; large framed and muscularly strong, yet giving one the impression of a person of weak will-power, easily controlled by appetite and deficient in visceral innervation. Three or four days previously the patient had been attacked with painful diarrhea and vomiting, for which he had received treatment without much result, except that the opiate in his medicine had temporarily controlled his pain, and afterward increased the nausea and general distress. Questioned with regard to his habits, the patient said he was temperate, but his notion of temperance was found to mean the use of four or five glasses of strong liquor daily, with an occasional spree. A little over-indulgence in alcoholics, combined with bad food, had precipitated this attack, to which his habits and lithemic condition predisposed.

On physical examination nothing was found wrong except a slight pharyngitis and the condition of the abdominal viscera. The stomach was distended with gas; the liver enlarged downward about three-quarters of an inch, and tender; there was quite uniform gaseous distension of the intestines, with moderate tenderness. At one point in the right hypochondrium, about four inches obliquely upward and to the right from the umbilicus, there was a small area of excessive tenderness, in which palpation detected a soft elevated something, apparently either a loop of intestine, or possibly part of an enlarged gall-bladder. There was moderate fever, the tongue was coated; the discharge from the bowels was offensive, consisting of soft, mud-colored lumps in dirty liquid. The urine was dark, depositing urates, but without albumin, casts or sugar. There was a slight sallowness, not amounting to jaundice.

The diagnosis was made of subacute parenchymatous hepatitis, gastro-enteritis, and possibly gall-stones. With regard to the condition of the liver, many would probably prefer to regard it as congestion. The existence of gall-stones was conjectural; none were found, and the passages of the first few days had already been thrown away. None of the later symptoms were significant in regard to this point.

Small doses of tincture of belladonna, fluid extract of cannabis

1. Read before the Buffalo Clinical Society.

indica, and carbolic acid were given through the afternoon and night, and the next day the patient was placed upon powders containing fifty centigrams each of salol and bismuth sub-carbonate, repeated every four hours. He was more than willing to accept my proposition to do away with opiates, although two or three nights later he took a quarter of a grain of morphine. The former physician-in-charge had prescribed a liniment containing a moderate amount of tincture of opium, and this I continued through the first night, partly on account of the relief of pain afforded,—an action mainly local, though to a considerable extent central, even when opium is used externally,—but largely because I make it a rule, in taking charge of a case which has been under the care of another physician, to continue, at least, some item of his treatment. There is a very widespread impression among the laity that a change of physicians means an entire upsetting of former methods of medication, and some members of the profession, unfortunately, are small enough to try to make capital for themselves by means of more or less open slurs upon the man whom they succeed. Leaving out of consideration the golden rule and the code of professional courtesy, the man who, by his speech and action, shows respect for his fellow-practitioners, not only raises the estimation of the whole medical profession in the minds of the laity, but gains for himself more credit than by insinuation or open criticism.

With reference to the treatment, carbolic acid is not an efficient antiseptic, either for internal or external use, unless its dose or strength be dangerous. It is, however, a good, though mild anesthetic, and by means of its slight antiseptic power it is especially indicated in painful conditions of the stomach with fermentation. Belladonna is also locally anesthetic, and it depresses the activity of involuntary muscle; hence its usefulness in painful affections of the stomach and bowel associated with excessive peristalsis. It would also have facilitated the passage of a gall-stone, had one been present. Cannabis indica acts in almost the same way as belladonna, although somewhat more mildly. Hence it may be used independently of, or auxiliary to, belladonna without danger of accumulating excessive amounts of atropine in the body. Cannabis indica has often proved disappointing, partly because too much has been expected of it, partly because too small doses have been given, partly because poor preparations have been used. In colicky conditions of the bowels, I like to use some form of menthol,—for example, the essence of peppermint. This particular patient objected to the taste of peppermint, and it was, therefore, not used. Bismuth subcarbonate is preferable to the subnitrate, because when decomposed a very weak acid gas, carbon dioxide, is evolved instead of the more active nitric residue. The difference, however, is more theoretical than practical.

Two days after my first visit, I introduced the stomach tube and washed out the stomach. This procedure caused extreme gagging and discomfort, but it was efficient in removing a considerable accumulation of mucus, bile and undigested food. For the first two or three days, almost no food except toast water was given. Later, broths, milk and bovinine were ordered. The patient was convalescent in four days and two days later he was discharged perfectly well, except for some slight weakness. On account of the patient's lithemic condition, it is quite probable that if his habits are indulged in, he will develop cirrhosis of the liver or kidneys, or perhaps of both. I have, therefore, advised him most earnestly to give up entirely the use of alcoholics.

